# Sleep problems and associated factors in a rural population of a Southern Brazilian city

**DOI:** 10.11606/S1518-8787.2018052000260

**Published:** 2018-09-13

**Authors:** Adriana Kramer Fiala Machado, Andrea Wendt, Fernando César Wehrmeister

**Affiliations:** I Universidade Federal de Pelotas. Faculdade de Medicina. Programa de Pós-Graduação em Epidemiologia. Pelotas, RS, Brasil; II Universidade Federal de Pelotas. Faculdade de Medicina. Departamento de Medicina Social. Pelotas, RS, Brasil

**Keywords:** Sleep Wake Disorders, epidemiology, Agrochemicals, adverse effects, Cross-Sectional Studies, Rural Population, Transtornos do Sono-Vigília, epidemiologia, Agroquímicos, efeitos adversos, Estudos Transversais, População Rural

## Abstract

**OBJECTIVE:**

To estimate the average of a sleep problems score and their associated factors in adults living in rural areas.

**METHODS:**

A population-based cross-sectional study with individuals from the rural area of the city of Pelotas, Southern Brazil. Twenty-four of the 50 census tracts that make up the eight rural districts of the city were randomly selected. Individuals of 18 years of age or older residing in the households chosen were considered eligible. Sleep problems were measured using the Mini Sleep Questionnaire, which ranged from 10 to 70 points and the higher the score, the greater the sleep problems. The independent variables evaluated included socioeconomic, demographic, behavioral and health characteristics. In the analysis, linear regression was used, obeying a previous hierarchical model.

**RESULTS:**

The sample consisted of 1,421 individuals. The average obtained for sleep problems was 29.4 points (95%CI 28.7–30.1). After adjusted analysis, the following variables were associated factors for greater sleep problems: female sex, age greater than or equal to 40 years, lower schooling level, depressive symptoms, pesticide poisoning, and poorer quality of life.

**CONCLUSIONS:**

The Mini Sleep Questionnaire average in this study was 4.4 points above the cut-off point that established sleep problems. The total points found in the score was high for the rural population. Strategies to improve sleep for these individuals should be focused on higher-risk groups such as women and the elderly and those with pesticide poisoning.

## RESUMO

**OBJETIVO:**

Estimar a média de um escore de problemas de sono e seus fatores associados em adultos residentes da zona rural.

**MÉTODOS:**

Estudo transversal de base populacional, realizado com indivíduos da zona rural do município de Pelotas, no Sul do Brasil. Foram selecionados aleatoriamente 24 dos 50 setores censitários que compõem os oito distritos rurais do município. Foram considerados elegíveis os indivíduos com 18 anos ou mais residentes nos domicílios sorteados. Os problemas de sono foram mensurados a partir do questionário *Mini Sleep Questionnaire*, cujo escore varia de 10 a 70 pontos e quanto maior a pontuação, maiores os problemas de sono. As variáveis independentes avaliadas incluíram características socioeconômicas, demográficas, comportamentais e de saúde. Na análise, utilizou-se regressão linear, obedecendo a modelo hierárquico prévio.

**RESULTADOS:**

A amostra foi composta por 1.421 indivíduos. A média obtida para problemas de sono foi de 29,4 pontos (IC95% 28,7–30,1). Após análise ajustada, as seguintes variáveis apresentaram-se como fatores associados para maiores problemas de sono: sexo feminino, idade maior ou igual a 40 anos, menor escolaridade, sintomas depressivos, intoxicação por agrotóxico e pior qualidade de vida.

**CONCLUSÕES:**

A média do *Mini Sleep Questionnaire* neste estudo foi 4,4 pontos acima do ponto de corte que estabelece problemas de sono. A pontuação total encontrada no escore foi elevada para a população rural. Estratégias para melhorar o sono desses indivíduos devem ser focadas em grupos de maior risco como mulheres e idosos e aqueles com intoxicação por agrotóxico.

## INTRODUCTION

Sleep disorders are recognized as a relevant public health problem, given the numerous consequences that they can bring to the physical and mental well-being of individuals^1^. These include increased fatigue, memory failure, attention and concentration difficulties, non-communicable chronic diseases, and use of substances such as alcohol, tobacco, and illicit drugs^2^. In addition, sleep disorders can increase the risk of work accidents and traffic accidents. In fact, one in five traffic accidents is attributed to excessive sleepiness when driving vehicles^2^.

In recent decades, the hectic lifestyle of societies has caused detrimental effects on individuals’ sleep^3^. This may explain, in part, the high prevalence of sleep disorders that are found in several countries with different levels of economic development. In the United States, it is estimated that between 25% and 30% of the adult population is affected by some decrease in sleep health^2^. In cities located in Mexico and in some South American countries (Uruguay, Chile, and Venezuela), snoring was the most prevalent sleep problem and was present in more than half of the population aged 40 years or older^4^.

In Brazil, a study carried out in 132 cities found that 76% of the sample, comprised of individuals aged 16 years or older, had at least one sleep problem, with the most frequent being insufficient sleep and snoring^5^. In São Paulo, the difficulty of initiating sleep in adults increased by 11% in the period between 1987 and 2007, with the prevalence being 25% in this last year^3^.

Although most studies on sleep-related problems are conducted in samples with urban residents, individuals living in rural areas also have a relatively high proportion of sleep-related problems. In Japan and China, respectively, 25.5% and 49.5% of rural individuals presented poor sleep quality^6^ ^,^ ^7^. In Brazil, we found only one population-based study that evaluated the theme in relation to the rural area, conducted with adolescents between 15 and 19 years old^8^. In this study, a significant increase in the prevalence of short-term sleep was observed between 2001 and 2011 (36.3% to 42.0%)^8^.

Results of research conducted in urban areas do not allow extrapolation to the rural area, given the markedly different characteristics of the environment, quality of life, work, and other behaviors^9^. Given the high prevalence of sleep problems found in urban areas, the absence of studies on the subject in the country’s rural adult population and its impact on health, the present study aimed to identify factors associated with sleep problems in adult residents of the countryside.

## METHODS

This research was carried out in the rural area of Pelotas, Rio Grande do Sul. The city is considered the third most populous of the state, with about 330,000 inhabitants, of which approximately 7% reside in the rural zone. Data from the Brazilian Institute of Geography and Statistics (IBGE) show that the rural population of Pelotas is predominantly male (51.4%), with individuals aged between 30 and 59 years old (41.7%)^10^. Data collection took place from January to July 2016, targeting individuals aged 18 years or older.

This is a cross-sectional population-based study, which is part of a larger survey investigating the health of the Pelotan population biennially since 1999, and this is the first one to include the city’s rural area^11^.

The rural area of the city has 50 census tracts, divided among eight districts. The sampling process in conglomerates occurred in two stages. First, 24 census tracts were selected, systematically, in proportion to the number of permanent residences in each district. Considering that there were two adults per household (IBGE), it was defined that 30 houses would be visited in each census tract. Subsequently, the Google Earth software was used, from which these sectors were divided into nuclei.

For the selection of nuclei, the site with the largest branch of streets was first found, which was called the center of the nucleus. The houses located within a radius of one kilometer of this center belonged to the same nucleus, which should contain at least five houses.

The selection of the residences began by the nucleus with the greater number of houses and, when arriving at the center of the nucleus, the direction to be traveled was drawn randomly. If all the residences were not identified in the first direction, we returned to the center of the nucleus and proceeded to the right-most path of the first. If a total of 30 residences were not reached in this nucleus, we moved towards the center of the second largest nucleus and the process was repeated. In each randomly selected household, we included all the residents that were 18 years of age or older. Exclusion criteria were institutionalized individuals at the time of the research and those with some cognitive impairment that made it impossible to answer the questionnaire.

To measure sleep problems, the Mini Sleep Questionnaire (MSQ)^12^, standardized and used in Brazil, was chosen^13^. This instrument consists of 10 questions, each with seven possibilities of answer ranging from never (one point) to always (seven points). Thus, the score ranges from 10 to 70 points and the higher the score, the more sleep problems there are.

The independent variables evaluated were: gender (male, female); skin color (reported by the interviewee and categorized as white, black or brown, others); age (18 to 24, 25 to 39, 40 to 59, 60 or more complete years); assets index, based on analysis of principal components, using household goods and possessions (categorized in quintiles); has partner (no, yes); schooling in full years (zero to four, five to eight, nine or more); and place of work (not working, urban area, rural area, both).

Total physical activity, measured by the Global Physical Activity Questionnaire (GPAC) [insufficiently active (< 150 minutes/week); active (≥ 150 minutes/week)] was also included as exposures; alcohol consumption, assessed by the instrument Alcohol use disorder identification test (AUDIT) (consumption without risk < 8 points; consumption with risk ≥ 8 points); current smoking (never smoked, ex-smoker, smoker, i.e., smokes every day, at least one cigarette, during the last month); pesticide poisoning, measured by the question “Have you ever had pesticide poisoning in your life?” (never, not sure, yes, certainly); quality of life, assessed using the question from the short WHOQOL questionnaire: “How is your quality of life?” (regular, bad, very bad, good, very good); and depressive symptoms assessed by the Edinburgh Postnatal Depression Scale (EPDS) (no symptoms < 8 points, shows symptoms > 8 points). The presence of morbidities was measured using the question, “Has a doctor or health professional ever told you that you have high blood pressure or blood sugar?”. Finally, weight and height were measured to calculate the body mass index (BMI), which was categorized according to the World Health Organization (WHO) criteria^14^ (≤ 24.9 kg/m^2^, 25.0 kg/m^2^ to 29.9 kg/m^2^, ≥ 30.0 kg/m^2^).

The information was collected by trained and standardized interviewers for the measurement of anthropometric data. The data was entered via tablets by the RedCap software.

The different aspects of sleep present in the questionnaire were evaluated by the proportions of each of their 10 questions. The answers were categorized into: never; very rarely or rarely; sometimes; often, very often or always.

Statistical analyzes were carried out using the svy command in Stata version 13.1 (Stata Corporation, College Station, USA), in order to consider the effect of cluster sampling. The sample was described by the proportion of individuals in each category of the variables, as well as the mean and the 95% confidence interval (95%CI) for the sleep problem score. The crude and adjusted analyzes were performed by linear regression and presented in coefficients (β) and 95%CI. For the adjusted analysis, a five-level hierarchical conceptual model was constructed, thus determining the order of input of the variables in the analysis. At the first level, the variables gender, skin color, and age were included; in the second level, living with a partner, schooling and assets index; at the third level, workplace; in the fourth level, physical activity, smoking, alcohol consumption and pesticide intoxication; and at the level most proximal to the outcome, depressive symptoms, quality of life, BMI, hypertension and diabetes. The stepwise regression model was used, in which all the variables of the first hierarchical level were introduced, and then those with p > 0.20 were excluded. The variables of the second level were adjusted for all those of the same level plus those of the previous level that remained in the model, repeating this procedure for the other levels. Factors associated with the outcome were considered as those with p < 0.05.

The project was approved by the Ethics Committee of the Faculdade de Medicina of the Universidade Federal de Pelotas (Approval 1.363.979). All participants signed the informed consent form.

## RESULTS

A total of 1,697 individuals were identified as eligible, of which 1,519 were interviewed, totaling 87 (5.1%) losses and 91 (5.4%) refusals. The average age between losses and refusals was 42.2 years (SD = 17.1), different from the one found in the sample, 47.8 (SD = 17.4, p = 0.007). The percentage of men among the losses and refusals was 70.8%, while in the sample, only 48.3% (p < 0.001). Sixty-six individuals (3.9%) did not respond to any question about the outcome and were considered as losses. Thus, information on sleep problems was obtained from 1,421 individuals, which is the final sample of the study.

The result of the interaction test for the sex variable was not significant. Table 1 shows the general characteristics of the sample and the average in the MSQ score according to each category of variables. The sample average in the score was 29.4 (95%CI 28.7–30.1). The individuals with the highest scores, that is, more sleep problems, were: female, aged between 40 and 59 years old, living with a partner, with less schooling, who did not work, who had poisoning by pesticide, with symptoms of depression, obese, hypertensive, diabetic, and with worse quality of life.

**Table 1:** Description of the sample and score averages of sleep problems in adults living in the rural area of Pelotas, State of Rio Grande do Sul, Brazil, 2016. (n = 1,421)

Variable	Total	MSQ	p^a^
			
	n (%)	Average (95%CI)	
Gender			< 0.001
Male	686 (48.2)	27.6 (26.8–28.5)	
Female	735 (51.8)	31.0 (30.2–31.8)	
Skin color			0.927
White	1,211 (85.0)	29.3 (28.6–30.1)	
Black or brown	181 (12.9)	29.4 (28.2–30.7)	
Others	29 (2.1)	30.0 (25.9–34.1)	
Age (years)			< 0.001
18–24	166 (11.7)	26.3(24.9–27.7)	
25–39	333 (23.6)	28.7 (27.6–29.8)	
40–59	561 (39.5)	30.3 (29.3–31.3)	
60 or older	361 (25.2)	30.0 (29.0–30.8)	
Romantic partner			0.002
No	400 (28.2)	28.0 (27.0–28.9)	
Yes	1,021 (71.8)	29.9 (29.1–30.7)	
Education (complete years)			< 0.001^b^
0–4	524 (37.1)	30.8 (29.7–32.0)	
5–8	528 (37.2)	29.3 (28.6–30.0)	
9 or more	364 (25.7)	27.4 (26.6–28.1)	
Assets index (quintiles)			0.440
5th (richest)	290 (18.8)	29.9 (28.4–31.4)	
4th	296 (19.9)	29.7 (28.4–30.9)	
3rd	282 (20.0)	30.0 (28.5–31.4)	
2nd	283 (20.8)	29.7 (27.8–30.0)	
1st (poorest)	260 (20.5)	28.9 (28.4–31.4)	
Workplace			0.001
Urban area	123 (8.7)	26.9 (25.6–28.3)	
Rural area	689 (48.4)	29.2 (28.1–30.2)	
Both	59 (4.1)	28.1 (26.0–30.3)	
Is unemployed	549 (38.8)	30.3 (29.4–31.2)	
Pesticide poisoning			< 0.001
Never	1,226 (86.5)	29.0 (28.3–29.7)	
Uncertain	109 (7.5)	31.8 (30.3–33.4)	
Certain	85 (6.0)	31.9 (30.0–33.9)	
Smoking			0.433
No	925 (64.8)	29.2 (28.2–30.1)	
Yes	229 (16.4)	30.0 (28.8–31.2)	
Former smoker	267 (18.8)	29.5 (28.5–30.4)	
Alcohol consumption			0.983
No risk	1,300 (94.0)	29.4 (28.7–30.0)	
At risk	121 (6.0)	29.4 (27.7–31.1)	
Physical activity			0.748
Active	1,157 (84.8)	29.3 (28.6–30.0)	
Inactive	208 (15.2)	29.5 (28.2–30.8)	
Depression (symptoms)			< 0.001
No	900 (64.5)	26.8 (26.2–27.4)	
Yes	492 (35.5)	34.1 (33.3–34.9)	
Nutritional status (BMI)^c^			0.030
Low weight/Eutrophic	475 (35.1)	28.8 (27.8–29.8)	
Overweight	480 (35.2)	29.2 (28.1–30.2)	
Obesity	404 (29.7)	30.2 (29.4–31.1)	
Quality of life			< 0.001
Very good	185 (13.0)	25.5 (24.3–26.8)	
Good	917 (64.5)	29.0 (28.1–29.8)	
Very bad/Bad/Fair	318 (22.5)	32.8 (31.8–33.7)	
Hypertension			< 0.001
No	946 (66.8)	28.2 (27.3–29.1)	
Yes	473 (33.2)	31.9 (30.9–32.9)	
Diabetes			< 0.001
No	1,282 (90.2)	29.1 (28.4–29.8)	
Yes	137 (9.8)	32.5 (31.0–34.0)	
Average Score (MSQ)	1,421 (100)	29.4 (28.7–30.1)	

MSQ: Mini Sleep Questionnaire; BMI: body mass index

^a^ Heterogeneity p-value.

^b^ Trend p-value.

^c^ Variable with the greater number of missing data (missings) (n = 1,359).

The figure shows the frequencies of each MSQ question according to sex. In general, the female sex reported more sleep problems. Using sedatives or sleeping pills very often, often or always had an occurrence about twice as high in women as in men, while snoring and sleeping during the day were reported more consistently by males.

**Figure:**
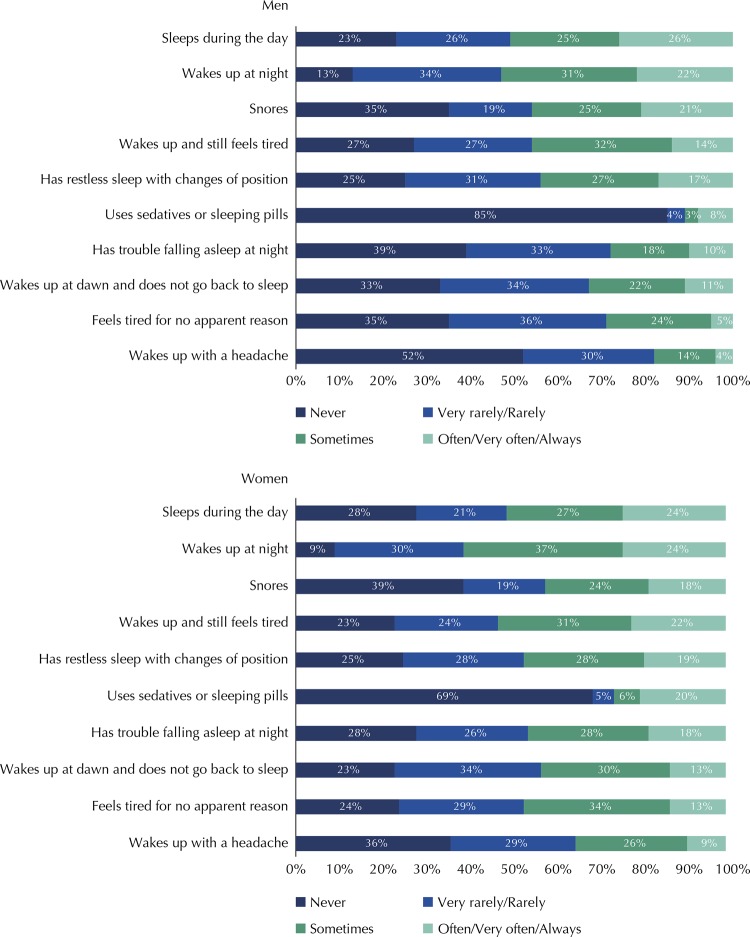
Frequency of Mini Sleep Questionnaire items, according to gender, in adults living in the rural area of Pelotas, State of Rio Grande do Sul, Brazil, 2016.

Table 2 presents the crude and adjusted coefficients of the association between sleep problems and independent variables. After an adjusted analysis, the women had a higher risk of presenting sleep problems, averaging 3.30 points higher in the score than the men. Those aged 40 years or older had more sleep problems than younger people (18–24 years). The schooling presented an inverse relation with sleep problems, i.e., individuals with lower schooling presented higher averages in the score. Among those who had pesticide poisoning, the score was, on average, 3.02 points higher than individuals who did not have it. People who presented symptoms of depression and reported regular, poor or very poor quality of life scored, on average, respectively, 5.19 and 4.05 more when compared to their counterparts. Hypertensive individuals had a score of 2.36 points, on average, higher than those without hypertension.

**Table 2:** Associations between sleep problems and independent variables in adults living in the rural area of Pelotas, State of Rio Grande do Sul, Brazil, 2016.

Level	Variable	Crude coefficient^a^		Adjusted coefficient^a^	

		β	p^b^	β	p^b^
1	Gender		< 0.001		< 0.001^c^
	Male	Ref		Ref	
	Female	3.33 (2.42–4.24)		3.30 (2.38–4.21)	
	Skin color		0.927		
	White	Ref			
	Black or brown	0.10 (-1.27–1.48)			
	Other	0.66 (-3.44–4.77)			
	Age (years)		< 0.001		< 0.001^c^
	18–24	Ref		Ref	
	25–39	2.39 (0.74–4.05)		2.26 (0.72–4.03)	
	40–59	4.00 (2.53–5.46)		3.91 (2.41–5.40)	
	60 or older	3.62 (1.91–5.33)		3.50 (1.83–5.18)	
2	Romantic partner		0.002		0.058^d^
	No	Ref		Ref	
	Yes	1.96 (0.80–3.13)		1.33 (-0.46–2.70)	
	Education (complete years)		< 0.001^e^		< 0.001^d,e^
	0–4	3.48 (2.25–4.72)		3.05 (1.68–4.42)	
	5–8	1.96 (0.97–2.96)		1.75 (0.73–2.77)	
	9 or more	Ref		Ref.	
	Assets index (quintiles)		0.440		
	5th (richest)	Ref			
	4th	0.38 (-1.51–2.26)		-	
	3rd	1.41 (-0.76–3.59)			
	2nd	1.12 (-0.50–2.75)			
	1st (poorest)	1.34 (-0.68–3.37)			
3	Workplace		0.001		0.206^f^
	Urban area	Ref		Ref	
	Rural area	2.25 (0.36–4.14)		1.02 (-0.75–2.79)	
	Both	1.22 (-1.45–3.88)		0.65 (-2.13–3.45)	
	Is unemployed	3.38 (1.90–4.86)		1.69 (0.12–3.25)	
4	Pesticide poisoning		< 0.001		< 0.001^g^
	Never	Ref		Ref	
	Uncertain	2.85 (1.30–4.39)		2.59 (1.08–4.10)	
	Certain	2.93 (0.68–5.17)		3.02 (0.97–5.08)	
	Smoking		0.433		
	No	Ref			
	Yes	0.85 (-0.47–2.16)		-	
	Former smoker	0.28 (-1.07–1.63)			
	Alcohol consumption		0.983		
	No risk	Ref		-	
	At risk	0.20 (-1.45–1.48)			
	Physical activity		0.748		
	Active	Ref			
	Inactive	0.20 (-1.05–1.44)		-	
5	Depression (symptoms)		< 0.001		< 0.001^h^
	No	Ref		Ref	
	Yes	7.32 (6.42–8.22)		5.91 (4.87–7.21)	
	BMI^i^		0.030		0.834^h^
	Low weight/Eutrophic	Ref		Ref	
	Overweight	0.39 (-0.89–1.66)		-0.27 (-1.29–0.76)	
	Obesity	1.46 (0.43–2.49)		-0.19 (-1.13–0.74)	
	Quality of life		< 0.001		< 0.001^h^
	Very good	Ref		Ref	
	Good	3.44 (1.89–4.98)		2.04 (0.65–3.44)	
	Very bad/Bad/Fair	7.26 (5.80–8.72)		4.05 (2.49–5.60)	
	Hypertension		< 0.001		< 0.001^h^
	No	Ref		Ref	
	Yes	3.70 (2.49–4.92)		2.36 (1.26–3.47)	
	Diabetes		< 0.001		0.080^h^
	No	Ref		Ref	
	Yes	3.45 (1.88–5.01)		1.15 (-0.15–2.44)	

BMI: body mass index; Ref: reference

^a^ Linear Regression.

^b^ Heterogeneity p-value.

^c^ Adjustments: sex and age.

^d^ Adjustments: sex, age, partner, and schooling.

^e^ Trend p-value.

^f^ Adjustments: sex, age, partner, schooling, and place of work.

^g^ Adjustments: sex, age, partner, schooling, and pesticide poisoning.

^h^ Adjustments: sex, age, partner, schooling, pesticide poisoning, depression, BMI, quality of life, hypertension, and diabetes.

^i^ Variable with greater number of missing (n = 1,359).

## DISCUSSION

The average sample score in the MSQ was high, 4.4 points higher than the limit determined by the instrument (25 points). Being female, 40 years of age or older, having less schooling, having been poisoned by pesticides, presenting depressive symptoms and reporting a normal, poor or very poor quality of life were the characteristics associated with more sleep problems.

It is possible that there is a direct relationship between higher scores in the MSQ score and worse health condition of individuals. According to Buysse^15^, sleep health is a multidimensional pattern of sleep-wakefulness, adapted to individual, social, and environmental demands, that promotes physical and mental well-being. Therefore, changes in its quality and duration may increase the risk of adverse effects such as morbidity and even mortality. We emphasize that there were no studies that used MSQ to evaluate the sleep of individuals living in rural areas; therefore, the comparison between the results should be done with caution. However, research using other methods have shown that sleep disorders affect a good part of this population^7^ ^,^ ^9^. In Japan, the mean score obtained on the Pittsburgh Sleep Quality Index (PSQI) in adults was 4.9 points; and the PSQI considers as sleep problems scores equal to or greater than five^16^. Li et al.^7^, using this same instrument, found an average score of 7.7 points in Chinese elderly. In Canada, both in urban and rural areas, 15.9% of those aged 18 years or older present excessive daytime sleepiness as measured by the Epworth Sleepiness Scale^17^. The studies found in the literature search are mostly from China^7^ ^,^ ^18^. The results are generally shown in the form of prevalence, which varies widely. These differences probably occur due to the lack of standardization in sleep measurements (quality, problem, insomnia or its symptoms, disorders), the different instruments used, the period evaluated (last month, last year, lifetime) and the different populations studied (adolescents, adults or elderly).

When assessing MSQ questions separately, we found that the most frequently reported situation in the sample was sleeping during the day. We observed that the sample studied awakens early in the morning, possibly due to work in the field, and returns to the house for lunch, remaining there until the climax of the heat decreases. Sleeping during the day, therefore, was set up as a habit in this population, not a behavior suggestive of sleep problems. In principal component analysis (PCA) (data not shown) including the 10 variables of the questionnaire, we verified that the MSQ score has a correlation coefficient of 0.96 (p < 0.001) with the score generated by the PCA. In addition, the behavior of sleeping during the day, even though the issue was reported more frequently, was the one that had less weight in the sum of the score (0.007).

In the present study, women presented worse average scores, which is consistent with the literature findings^18–20^. A multicenter study with urban and rural data from low- and middle-income countries showed that men are 23% less likely to present sleep complaints^19^. Hormonal, behavioral, and psychological factors may be responsible for this association. In younger women, biological factors such as motherhood can adversely affect sleep, whereas in later ages, menopause may lead to worsening of the sleep quality^21^. In addition, anxiety and depression, known to be more frequent in females, may also be present in this relationship^21^.

It is widely reported in the literature that the occurrence of sleep problems tends to increase with age^7^ ^,^ ^18^ ^,^ ^19^ ^,^ ^21^. However, in this study, this trend was not observed, considering that the risk of presenting the outcome was not different above 40 years of age. It is possible that the elderly did not present the worst result due to the occurrence of survival bias, in which older individuals with poorer sleep quality and a greater burden of chronic diseases did not participate in the study since they have already died. Another factor that could lead to the observed result is the possibility that sleep perception may be different in the elderly, which may result in fewer complaints^22^.

The findings on the association between sleep problems and educational level in the literature are inconclusive. In middle- and lower-income countries, having a higher education level reduced the risk of complaining of sleep problems by 6%; in China, presenting lower levels of education reduced the risk of snoring and apnea by 27%. Nonetheless, other studies did not find associations^18^ ^,^ ^21^. However, lower schooling leads to manual occupational activities and long hours of work, which may contribute to an increase in the occurrence of sleep disorders^9^. In addition, a higher educational level is associated with health-promoting behaviors, thus preventing events that may cause sleep problems^9^.

There are numerous consequences of the use of pesticides on human health, among them neurobehavioral alterations^24^. Central nervous system dysfunctions due to the use of these pesticides can lead to insomnia or disturbed sleep, excessive dreams or nightmares^24^. In China, researchers have observed a higher occurrence of excessive daytime sleepiness among those who have been exposed to organophosphates for longer^25^. In European countries, exposure to pesticides was a potential risk factor for REM (rapid eye movement) disorders^26^.

The literature has suggested that leisure physical activity promotes sleep quality improvement^19^, but this relationship was not found in our study. This may have occurred due to the fact that almost all the participants performed physical activity only at work, and physical activity was practically non-existent. Physical activity in leisure has been associated with better health conditions and, consequently, better sleep^19^. Excessive physical activity at work can be detrimental to health and sleep. Akerstedt et al.^27^ argue that both the high demands of work and the physical effort performed in this environment have a negative impact on sleep.

The association between the presence of depressive symptoms and increased sleep problems found in this study is consistent with the literature^18^ ^,^ ^19^ ^,^ ^21^. Serotonin, the key hormone in sleep regulation, is decreased during a depressive episode, often leading to insomnia. On the other hand, changes in the circadian rhythm, such as changes in usual sleeping time and sleep deprivation, may induce depressive symptoms. Therefore, changes in sleep may occur before the depressive episode, in its early stages, or may be a residual symptom of non-responsive depression^28^.

The relationship between sleep problems and quality of life found here is also described by other investigations. In China, a study of individuals between 15 and 34 years old found worse quality of life in those with insomnia or sleep disorders^18^. The possible mechanism for this association is that problems in sleep can affect the ability of the immune system to function properly, impair performance in school, work and social activities, as well as have a great physical, mental and emotional impact on overall quality of life^29^. This relationship may also occur indirectly, since poorer sleep quality is associated with the development of chronic diseases, such as hypertension and diabetes^20^ ^,^ ^30^, which in turn may have a negative impact on quality of life^29^.

Hypertensive individuals presented greater sleep problems: the relationship between these variables is bidirectional. Sleep deprivation can affect the sympathetic nervous system and alter the secretion of cortisol and antidiuretic hormone, increasing blood pressure^31^. On the other hand, the lower cerebral blood flow caused by hypertension can be offset by increased flow in other areas of the brain and these changes over time can manifest as sleep disorders^32^. Furthermore, the use of diuretics to control blood pressure intensifies the need to urinate, which can increase the fragmentation of sleep.

In this study, we did not find an association of sleep quality with alcohol consumption, smoking, and BMI. In a complementary analysis (data not shown), we could observe that the occurrence of snoring was greater in those who were obese and smokers, but this association was not maintained when we evaluated the questionnaire as a whole. These factors seem to be more related to respiratory sleep disorders than to their quality^23^ ^,^ ^32^. Regarding alcohol consumption, some studies have shown that this relationship seems to depend on the number of doses ingested and the stage of use^33^.

When interpreting the results presented here, one should consider some limitations of the study. The cross-sectional design does not allow us to infer causality in some associations, such as depression, quality of life, and hypertension, considering the bidirectional relationship that these variables may present with sleep problems. However, this outline has the potential to raise hypotheses and subsidize public health policies. The absence of information regarding the type, the workload, and the work shift did not allow for a more detailed analysis of its relationship to the outcome. The percentage of losses and refusals obtained (14.4%) should be noted: these individuals presented characteristics (greater percentage of men and youngest individuals) different from the sample. This may suggest an overestimation of the results, since losses and refusals include individuals who, according to the literature^18^ ^,^ ^19^ ^,^ ^21^, have fewer sleep problems. In addition, the questionnaire used by this study is not validated for rural populations. As rural dwellers present different characteristics and behaviors when compared to the urban population, the questions present in the instrument may not reflect the real distribution of the sleep problems of this sample. However, a pilot study was conducted to verify the understanding of the questionnaire and to minimize this possible limitation.

Aspects related to sleep are rarely studied in rural areas, be it in Brazil or in the world. The MSQ average in this study was 4.4 points higher than the cutoff point that established sleep problems. The associated factors were female gender, age of 40 years or older, less schooling, pesticide poisoning, depressive symptoms, hypertension, and poorer quality of life. A better understanding of the dimension of the aspects involved in other communities is essential to better understand and cope with the problem.
